# DNA Methylation Array Analysis Identifies Biological Subgroups of Cutaneous Melanoma and Reveals Extensive Differences with Benign Melanocytic Nevi

**DOI:** 10.3390/diagnostics15050531

**Published:** 2025-02-21

**Authors:** Simon Schwendinger, Wolfram Jaschke, Theresa Walder, Jürgen Hench, Verena Vogi, Stephan Frank, Per Hoffmann, Stefan Herms, Johannes Zschocke, Van Anh Nguyen, Matthias Schmuth, Emina Jukic

**Affiliations:** 1 Institute of Human Genetics, Medical University Innsbruck, 6020 Innsbruck, Austria; simon.schwendinger@i-med.ac.at (S.S.);; 2 Department of Dermatology, Venereology and Allergy, Medical University Innsbruck, 6020 Innsbruck, Austria; 3 Institute of Medical Genetics and Pathology, University Hospital Basel, 4031 Basel, Switzerland; 4 Institute of Human Genetics, University Hospital Bonn, 53127 Bonn, Germany

**Keywords:** cutaneous melanoma, melanocytic nevi, genetics, epigenetics

## Abstract

**Background/Objectives**: Genetics and epigenetics play an important role in the pathogenesis of cutaneous melanoma. The majority of cases harbor mutations in genes associated with the MAPK signaling pathway, i.e., *BRAF*, *NRAS*, or *NF1*. The remaining neoplasms, often located on acral sites, are condensed as the triple-wildtype subtype and are characterized by other molecular drivers. This study aimed to elucidate genetic and epigenetic differences within cutaneous melanoma and to compare it with melanocytic nevi. **Methods**: DNA was extracted from archived tissue samples of cutaneous melanoma (*n* = 19), melanocytic nevi (*n* = 11), and skin controls (*n* = 11) and subsequently analyzed by massive parallel (next generation) gene panel sequencing and genome-wide DNA methylation array analysis. The sample size was increased by including repository data from an external study. **Results**: There were major differences in the genomic landscape of MAPK-altered and triple-wildtype cutaneous melanoma, the latter presenting with a lower number of mutations, a different pattern of copy number variants, and a low frequency of *TERT* promoter mutations. Dimensional reduction of DNA methylation array analysis clearly separated cutaneous melanoma from melanocytic nevi but revealed no major differences between classical cutaneous melanoma and the triple-wildtype cases. However, it identified a possible biological subgroup characterized by intermediately methylated CpGs. **Conclusions**: Dimensional reduction of methylation array data is a useful tool for the analysis of melanocytic tumors to differentiate between malignant and benign lesions and may be able to identify biologically distinct subtypes of cutaneous melanoma.

## 1. Introduction

Cutaneous melanoma (CM) is characterized by activating mutations in the MAPK signaling pathway, including *BRAF* mutations in 50% of cases, *RAS* alterations in about 30% of cases, and *NF1* deficiencies in 15% of the neoplasms [1,2]. CMs lacking these common mutations have been classified as the triple wildtype (TWT) subtype. This group of neoplasms is very heterogeneous, and different molecular drivers, such as *KIT* gene mutations, seem to play a role [2]. TWT-CMs are often located at acral sites. TWT-CMs, in general, and acral CM, in particular, are usually not associated with sun damage and have fewer genetic alterations than classical MAPK-activated CM [1]. The most common subtype of acral CM is acral lentiginous melanoma (ALM), which has a characteristic lentiginous growth pattern. Rarer manifestations at acral sites are nodular or superficial spreading CMs [3,4].

Epigenetic alterations play an important role in the development of CM. The DNA methylation pattern (methylome) of CM is characterized by global hypomethylation combined with local hypermethylation. Both mechanisms contribute to tumorigenesis by activation of oncogenic factors and suppression of tumor suppressor genes [5,6]. Various studies have found that the methylation status of CM changes during disease progression [7,8]. In particular, gene promoter hypermethylation increases with CM progression. A high degree of promoter methylation is referred to as CpG island hypermethylation phenotype (CIMP) and is associated with poor clinical outcomes [7,8,9,10,11].

Our present study aimed to investigate the genetic and epigenetic landscape of CM in comparison to melanocytic nevi (MN). Regarding methodology, the study is based on massive parallel sequencing (next-generation sequencing; NGS) and genome-wide DNA methylation array (DMA) analysis. Apart from CM with classical driver genes, a high number of TWT cases (many of them from acral sites) are included in the study to characterize the genetics and DNA methylation patterns in this rare CM subtype. We examine whether methylome analysis can distinguish CM from MN and identify differences between MAPK-altered CM and TWT-CM in relation to genetic markers. Additionally, we analyze epigenetic differences between MN and the two CM subgroups based on differentially methylated positions (DMPs) and regions (DMRs).

## 2. Materials and Methods

### 2.1. Patients and Samples

A cohort was generated from formalin-embedded paraffin (FFPE) blocks derived from primary CM tumors (*n* = 56) with a Breslow’s thickness ≥ 1.8 mm. Additionally, MN (*n* = 56) with >3 mm diameter and healthy skin samples (*n* = 11) were included. CM patients from the time period between 2006 and 2021 were identified in the Tumor Registry Tyrol. Written informed consent was obtained under two protocols approved by the Ethics Committee of the Medical University of Innsbruck (No. 1182/2018 and 1170/2019). Clinical information, including sex, age at diagnosis, tumor location, tumor stage, the morphological classification of the primary tumor at diagnosis, as well as histological subtype and Breslow’s thickness, were recorded. The cases were re-evaluated by a dermatopathologist to confirm the diagnosis and morphological classification. All cases were retrieved from residual tissue blocks from the dermatopathology archives of the Medical University of Innsbruck and cooperating institutes. Analyses were performed after de-identification of the specimen. Additional data for samples from an external study published by Pradhan et al., 2019 [4] were downloaded from the Gene Expression Omnibus [12]. This dataset includes CM samples (*n* = 40) and MN specimens (*n* = 3), most of them localized in acral skin.

### 2.2. Macrodissection and DNA Isolation

Serial sections were prepared from FFPE tissue blocks. A hematoxylin and eosin (H&E)-stained reference slide was prepared from the central section. This specimen was assessed by a dermatopathologist, and tumor areas were marked. The marks were transferred to adjacent unstained sections. A standard xylol–ethanol protocol was used to remove paraffin from the samples, and a manual macrodissection of the sections was performed. After incubation with proteinase K, genomic DNA was isolated using the QIAmp DNA FFPE tissue kit (Qiagen, Hilden, Germany) according to the manufacturer’s instructions. The DNA was quantified using a micro-volume photometer and the Qubit 4 fluorometer with the dsDNA High Sensitivity or dsDNA Broad Range assay kits (Thermo Fisher Scientific, Waltham, MA, USA).

### 2.3. Methylation Array Analysis

#### 2.3.1. Array Preparation and Scanning

For methylation array analysis, the Illumina Infinium MethylationEPIC BeadChip Array platform (Illumina, San Diego, CA, USA) was used. Depending on the available DNA quantity, between 150 and 250 ng of DNA was utilized per sample. The specimens were prepared with the standard protocol for FFPE materials according to the manufacturer’s instructions and scanned on an iScan device (Illumina, San Diego, CA, USA).

#### 2.3.2. Quality Control and Tumor Purity Estimation

For quality control and tumor purity estimation, the IDAT files containing the methylation data were loaded into R Studio (version 1.4.1717; R version: 4.1.0) using the minfi [13] package (version 1.40.0). For every probe on the array, the detection *p*-value (detp) was calculated. As the share of failed probes (i.e., a detp > 0.01) can be used as a surrogate marker for array data quality [14], all samples with more than 5% of failed probes were excluded from the dataset. To estimate the tumor cell content of the CM and the MN samples, the RFpurify [15] package (version 0.1.2) was used. In brief, this method uses the methylation status of 856 CpGs to estimate the ABSOLUTE and ESTIMATE values of a sample by random forest regression and, therefore, predicts the tumor purity of the sample [15]. A careful evaluation in synopsis with the subsequently described UMAP analysis showed that a predicted ABSOLUTE score of below 0.445 or a predicted ESTIMATE value of below 0.775 seemed to work best for excluding samples with low tumor purity to achieve clearly separated sample clusters. Samples that failed the prediction cutoffs but were still localized in one of the sample clusters were included again to increase the sample size.

#### 2.3.3. Uniform Manifold Approximation and Projection (UMAP) Analysis

Methylome analysis was performed by uniform manifold approximation and projection (UMAP). This method reduces the highly dimensional methylation array datasets down to two dimensions. The EpiDiP server of the University Hospital, Basel, Switzerland (available from http://s1665.rootserver.io/, accessed on 15 April 2022) was utilized. The total dataset for dimensional reduction includes about 25,000 datasets from various cancer samples derived from publicly available data repositories such as The Cancer Genome Atlas [16] and the Gene Expression Omnibus [12], as well as thousands of samples derived from various contributors. UMAP reduction was performed using the 50,000 probes with the highest standard deviations among all samples. For the presented two-dimensional plots, only the coordinates from the samples of the current project were extracted.

#### 2.3.4. Differential Methylation Analysis

For DMP and DMR analysis, a strategy based on a protocol published by Maksimovic et al., 2016 [14] was applied. Normalization was performed using the functional normalization method in minfi. Subsequently, all probes with a detp > 0.01 in at least one sample, probes containing a SNP in their sequence, and all probes mapping to sex chromosomes were excluded. Further, probes known to be cross-reactive were filtered using the maxprobes package (version 0.0.2; available from https://github.com/markgene/maxprobes, accessed on 28 April 2022). After filtering, 678,635 CpGs remained in this pipeline. DMP analysis between the methylation clusters was performed using a linear model design with group-wise comparisons and empirical Bayes statistics from the limma package (version 3.50.3) [17]. The same model was subsequently used for the identification of DMRs with the DMRcate package (version 2.8.5) [18]. Regions comprising at least five CpGs and a mean methylation difference of 0.25 were called. The top ten significant DMRs from all three comparisons were evaluated using the UCSC genome browser [19] with the tracks “RefSeq Transcripts” and GeneHancer [20] (double elite) promoters and enhancers displayed.

### 2.4. Copy Number Variant (CNV) Detection

Copy number variant (CNV) profiles were calculated using the methylation array data and the conumee package (version 1.9.0; available from http://bioconductor.org/packages/conumee, accessed on 30 April 2022) in R Studio. The method uses the total intensities (red and green) of every probe for calculation, segmentation, and calling of differences in copy numbers of chromosomal regions. The analysis was conducted according to the instructions in the package vignette. In short, samples were loaded and normalized using the quantile normalization method of the minfi package version 1.40.0 [13]. For the actual analysis, a minimum number of 30 probes per bin and a minimum bin size of 100,000 bp were chosen. Genes of interest were chosen according to several publications and our personal interests. Because of the limited data quality for CNV analysis from FFPE-derived DNA, automated CNV calling was not expedient (too many false positives or false negatives due to strong scattering of log2 ratio values). Instead, CNVs were identified by careful manual inspection of the genome plots produced by the package. For this, the plots were inspected for segments of chromosomes with deviations in the log2 ratio of at least 0.3–0.4.

### 2.5. Next Generation Sequencing

#### 2.5.1. Library Preparation and Sequencing

Enriched sample libraries for sequencing were prepared using an in-house pipeline based on the Illumina DNA Prep with Enrichment kit and a custom-designed hybridization probe panel (Illumina, San Diego, CA, USA). A total of 100–250 ng of DNA was used per sample. The utilized probe panel includes selected exons or full coding regions of about 153 genes with a total covered region of 0.49 mbp. Detailed information about the covered regions is provided in Supplementary Table S1. Paired-end sequencing was performed on an Illumina NextSeq 500 device (Illumina, San Diego, CA, USA). The mean target coverage depth of the samples was 936 ± 451 SD reads, with a mean of 99.78% ± 0.32% SD of the targeted regions covered at least 50-fold. For FASTQ generation and data processing, the Illumina BaseSpace Sequencing Hub (BSSH; European instance in Frankfurt, Germany; available from https://euc1.sh.basespace.illumina.com, accessed on 3 October 2021) was utilized. Alignment of raw reads and variant calling was performed with the DRAGEN Enrichment app v3.6.3, with hg19 as the reference genome. Variants with a variant allele frequency (VAF) of at least 10% and a quality score of 20 were called. Only CM and MN samples were analyzed; no sequencing was performed for the skin control samples.

#### 2.5.2. Small Variant Evaluation

The called variants were annotated to RefSeq transcripts using the Illumina annotation engine (Illumina, San Diego, CA, USA) in BSSH Variant Interpreter (European instance in Frankfurt, Germany; available from https://variantinterpreter.euc1.vi.basespace.illumina.com, accessed on 3 October 2021). All datasets were filtered for coding variants and splice site variants. As no matched normal samples were available, the exclusion of probable germline variants was performed by filtering polymorphisms with a minor allele frequency of above 0.01 in the non-Finnish European population data in the Genome Aggregation Database (GnomAD) [21]. The remaining variants were verified by manual evaluation of alignments with the Integrative Genomics Viewer (IGV) [22]. Some variants were excluded during this process because they were identified as sequencing artifacts. As a last step, variants were carefully evaluated using the Catalogue of Somatic Mutations in Cancer (COSMIC) [23] as well as the ClinVar [24] and the My Cancer Genome [25] databases. Variants with prevailing evidence of germline origin were excluded from the dataset; the other variants were classified as pathogenic, likely pathogenic, or unclear significance based on their database presence or predicted functionality using SIFT [26] and PolyPhen2 [27]. *TERT* promotor analysis was performed by manual evaluation of alignments using IGV. Variants were manually annotated to the RefSeq transcript NM_198253.3. According to Nagore et al., 2019 [28] and Hugdahl et al., 2018 [29], samples were scoured for the variants c.-57A>C, c.-124C>T, c.-124/-125CC>TT, c.-138/-139CC>TT, and c.-146C>T.

### 2.6. Data Evaluation and Statistics

All statistical analyses were performed using R. During differential methylation analysis, hundreds of thousands of parallel hypothesis tests are conducted; therefore, strategies to control for false positive results are routinely implemented for both DMP and DMR discovery. For DMP identification, the limma package uses a significance level of α = 0.05 and adjusts all *p*-values using the Benjamini–Hochberg method [30], with an accepted false discovery rate of 5% [17]. DMRcate additionally uses a kernel smoothing approach to average test statistics across adjacent CpG sites, reducing variability in individual tests and improving the detection sensitivity of DMR identification [18].

During data evaluation, different statistical tests were performed to compare groups, depending on the specific hypothesis. In summary, the Wilcoxon rank-sum test, Fisher’s exact test, or analysis of variance (ANOVA) followed by pairwise t-tests were applied. The non-parametric tests were chosen because sample sizes for the corresponding research questions were relatively small. When necessary (i.e., when two or more parallel tests were conducted within one comparison of groups), *p*-values were adjusted using the Benjamini–Hochberg method [30]. The test used for calculation is indicated with each individual *p*-value.

## 3. Results

In this study, FFPE tissue blocks of primary CM or MN were analyzed. For some samples, the absence of a *BRAF* or *NRAS* hotspot mutation was known from a preceding study [31] or from routine diagnostic testing. Such cases were preferably chosen to ensure a high proportion of cases with TWT status. All samples were manually macrodissected and DNA was isolated from the tumor fraction. In total, 56 samples from both groups were used; however, the majority of samples had to be excluded due to technical reasons or low tumor purity (i.e., low content of tumor cells). A complete list of all analyzed samples with additional metadata and (if applicable) exclusion reason is provided in the Supplementary Data File. Supplementary Figure S1 visualizes the age of FFPE blocks for included and excluded samples. The median block age of excluded samples was significantly higher than the included ones both for MN (Wilcoxon test *p*-value: <0.001) and CM (Wilcoxon test *p*-value: 0.005). Evaluation of biological parameters of CMs such as tumor area, Breslow’s depth, and cell vitality did not reveal clear differences between included and excluded samples. However, the inclusion rate was much higher in tumors presenting ulceration, and all included samples were of tumor stage IIB or higher; these comparisons are visualized in Supplementary Figure S2.

The final cohort consisted of 19 CM and eleven MN specimens. As reference material, eleven skin samples from healthy donor skin were included. A list of the samples in the final cohort with all collected biological as well as clinical parameters is provided in the Supplementary Data File; an aggregated overview of the cohort can be seen in Table 1. In summary, the majority of the included CM samples were derived from acral or extremity localization and presented with nodular histology, advanced tumor stage, and a high Breslow’s depth.

### 3.1. Genetic Analysis Reveals Distinct Differences Between Cases with Alterations in the MAPK Signaling Pathway and TWT Status

**Figure 1:**
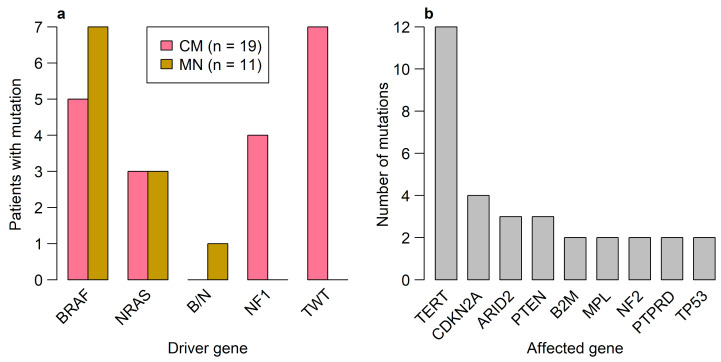
Driver mutations in CM and BN cases and mutational landscape of CM patients. (**a**) Bar plot depicting the proportion of patients with a MAPK-associated driver mutation (pink = CM, orange = MN; B/N = BRAF and NRAS mutations) for both CM and BN. (**b**) Bar plot showing the absolute frequencies of genes with a pathogenic or likely pathogenic mutation in the overall CM cohort. Only genes that were recurrently mutated (i.e., at least twice) are shown.

Genetic analyses of CM samples by NGS of approximately 150 genes revealed—as expected due to the selection bias of specimens—that most of the cases (7/19; 37%) had a genetic TWT status (Figure 1a). *BRAF* mutations (exclusively p.V600E or p.V600K) were found in 5/19 samples (26%), *NF1* mutations were found in 4/19 (21%) samples, while 3/19 (16%) samples harbored an *NRAS* mutation (Figure 1a). In addition to the established driver mutations, the most common alterations classified as pathogenic or likely pathogenic were *TERT* promoter mutations in 12/19 (63%) cases, followed by mutations in *CDKN2A* in 4/19 (27%) cases, and *ARID2* and *PTEN* mutations in 3/19 (16%) cases.

An overview of the most recurrently mutated genes is shown in Figure 1b. The complete list of all detected mutations, including information used for classification, is provided in the Supplementary Data File. The mean number of mutations per sample (including variants of unclear significance) was 5.3 ± 4.8 standard deviation (SD). Samples with one of the three driver mutations had a significantly higher mean mutation count of 5.3 ± 3.1 SD compared to 4 ± 3.2 SD in the TWT group (Wilcoxon test *p*-value: 0.005). *TERT* promoter mutations were found in 10/12 (83%) cases with a MAPK signaling pathway activating driver mutation but only in 2/7 (29%) of samples with TWT status (Fisher’s exact test *p*-value: 0.009). In accordance with De Martino et al., 2020 [31], no apparent connection between genetics and altitude of residence of the patient was observed. In the remaining sample, both *BRAF* p.V600E and *NRAS* p.Q61K mutations were detected.

Apart from the driver mutations, only a few mutations were detected in MN cases, with a mean mutation count of 2.4 ± 1.3 SD. None of the MN samples harbored a *TERT* promoter mutation.

**Figure 2:**
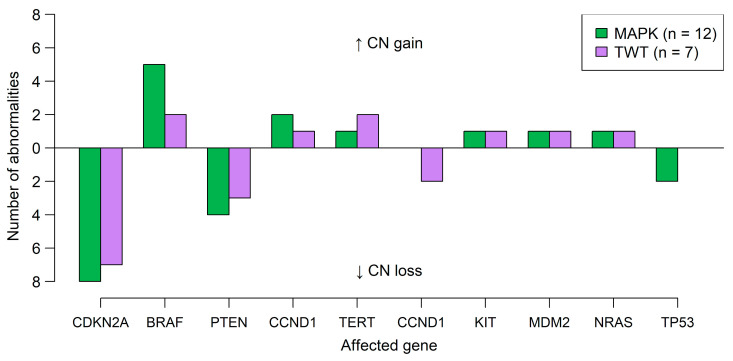
Cytogenomic landscape of CM samples. Frequencies of CNVs detected in MAKP-altered CM (green) and TWT (violet) cases. CNVs are shown as mirrored bar plots, with gains of chromosomal material oriented to the top and losses displayed on the bottom.

Data obtained from the DMA analysis were used for the analysis of CNVs. Due to the limited quality of FFPE-derived DNA, we generated CNV plots and identified extensive CNVs by manual inspection of the plots (see Section 2). The CNV plots of all samples and a list of the detected alterations, as well as a general assessment of the CNV pattern profile (i.e., widespread or more focal changes), are provided in the Supplementary Data File. Figure 2 summarizes CNVs in the two sample groups affecting certain genes considered relevant for CM development. For MAPK-CM, the CNV patterns showed mainly extensive abnormalities involving large parts of chromosomal arms or whole chromosomes, consistent with reports from the literature [32]. The most commonly deleted regions included 9p21 (*CDKN2A* locus) in 8/12 (67%) and 10q23 (*PTEN* locus) in 4/12 (33%) samples, whereas the most prevalent amplified region was 7q34 (*BRAF* locus) in 5/12 (42%) samples. Similar CNV patterns were observed in most TWT samples; however, these samples had a tendency for more focal amplifications or deletions. Loss of the *CDKN2A* locus appeared in 7/7 (100%) of the samples, and deletions including the *PTEN* locus were observed in 3/7 (33%) samples. Recurrently amplified regions were again the *BRAF* locus on 7q34 and the *TERT* region on 11q13 in 2/7 (33%) of cases. One TWT sample (in0597) presented a distinct, generally flat CNV profile with only a few but very complex focal abnormalities. The latter sample was derived from an acral desmoplastic melanoma and did not show any mutations except an in-frame deletion in a gene called *SOCS1*. None of the MN samples harbored CNVs.

### 3.2. DNA Methylome Analysis Identifies Differences Between CM and MN and Indicates a Distinct Biological Subtype of CM

To assess the general differences in the methylome structures of CM, MN, and skin samples, a DMA analysis was performed and the dimensional reduction method UMAP was applied. This method reduces the highly complex datasets down to two dimensions, which can be plotted and viewed. Samples with a similar methylome are located near each other, whereas distinct samples are separated into individual clusters [33]. In the chosen approach, the samples are evaluated within a big data lake consisting of about 25,000 different tumor samples [34], and the coordinates of the relevant specimens are extracted afterward. A depiction of the reduction can be seen in Supplementary Figure S3. It resulted in three distinct groups of sample clusters (termed methylation clusters) comprising all but one of the CM specimens (termed melanoma methylation cluster 1; MMC1), MN samples (nevus methylation cluster; NMC), and healthy skin controls (skin methylation cluster; SMC). One CM sample (in0597) was localized separately from the other samples. This sample is of particular interest, as it shows a methylome structure different from all other CM cases. Intriguingly, this sample is also the one described before, harboring a distinct CNV pattern and TWT genetic status.

**Figure 3:**
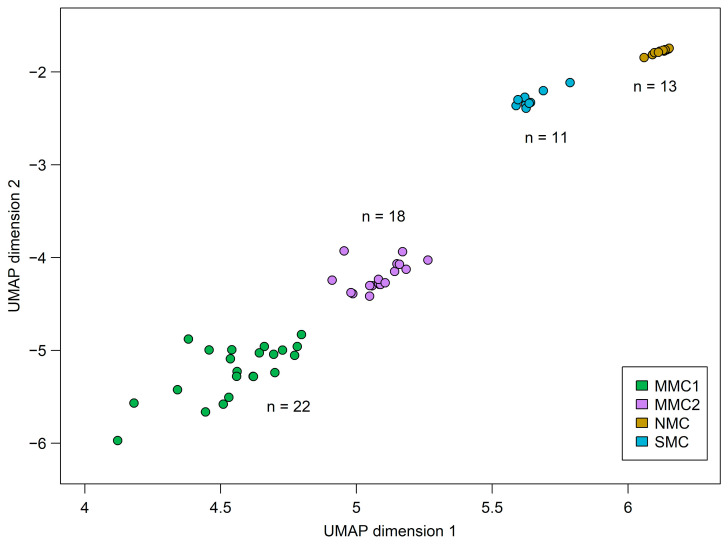
Methylation clusters determined by UMAP reduction analysis. Two-dimensional UMAP reduction plot of all samples from the internal and external cohorts. Each dot represents the overall methylome structure of one sample. UMAP reduction was performed on the EpiDip server together with approximately 25,000 other cancer and normal samples. The coordinates of the specimens in this study were subsequently extracted. The individual methylation clusters are classified as melanoma methylation cluster 1 (MMC1; green), melanoma methylation cluster 2 (MMC2; magenta), nevus methylation cluster (NMC; orange), and skin methylation cluster (SMC; blue).

To see whether this result can be reproduced with additional samples, we searched the Gene Expression Omnibus [12] for publicly available methylation datasets and found a promising study published by Pradhan et al. in 2019 [4] focusing on the epigenetics of ALM and other acral CMs. We processed those samples with our quality control pipeline and analyzed the remaining 21 CM specimens and two MN samples with our cohort. As shown in Figure 3, 17 of those specimens clustered together with our in0597 sample and comprised a second methylation cluster (melanoma methylation cluster 2; MMC2) separated from the MMC1 cluster. The UMAP coordinates used to produce the plot, as well as the cluster designation of each individual sample, are provided in the Supplementary Data File. According to the available metadata, most of the samples located in MMC2 were derived from primary ALM specimens; additionally, three samples classified as non-ALM acral CM and two samples classified as CM were located in this cluster. The dataset also added four samples of mixed histology to our MMC1 cluster and contained two acral MN samples that co-clustered with the NMC cluster.

**Figure 4:**
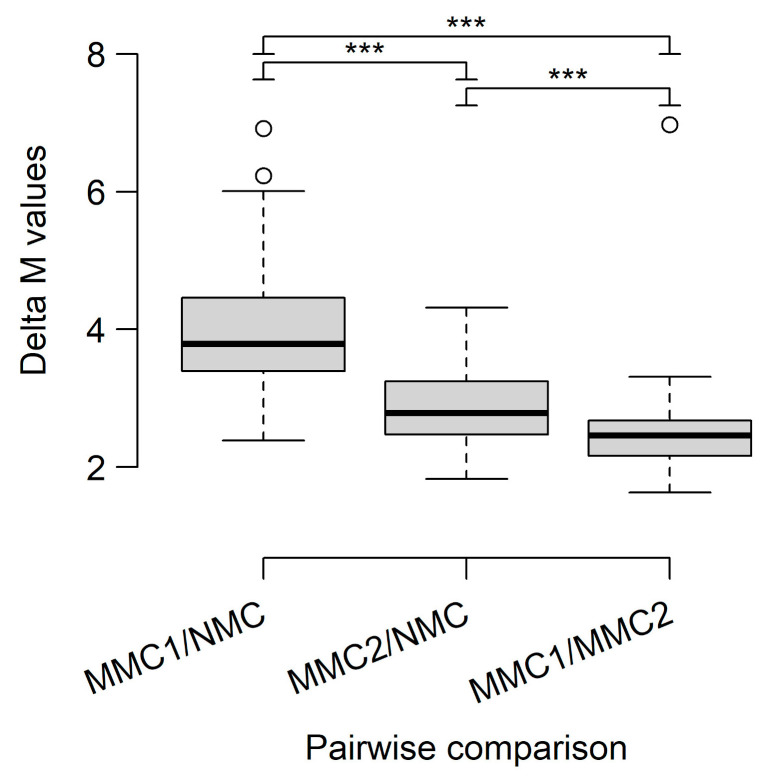
Median differences in M-values for pairwise DMA. The differences in the M-values of the top 100 significant CpGs from each of the three comparisons during DMP analysis were calculated. Their distributions are shown as Box-and-Whisker plots, with the box representing the median and interquartile range (IQR), whiskers extending to 1.5-times the IQR, and circles indicating outliers. Significance testing between the three comparisons was performed with ANOVA and pairwise *t*-tests, with *p*-values adjusted using the Benjamini–Hochberg method. The three stars (***) depict that for all three pairwise comparisons, the adjusted *p*-value was far below 0.001.

We then used the data from both cohorts together to assess the DMPs and DMRs between MMC1, MMC2, and NMC. For this, we used an analysis pipeline with group-wise comparisons based on a protocol published by Maksimovic et al., 2016 [14]. This approach identified 25.3% CpGs as significantly differentially methylated between MMC1 and NMC, 27% for MMC2 vs. NMC, and 23.9% for MMC1 vs. MMC2. A list of the top 100 significant DMPs for all three comparisons, with appropriate metadata, is provided in the Supplementary Data File. While the comparison between MMC1 and NMC resulted in DMPs with a high difference in methylation status, both comparisons with MMC2 showed mainly CpGs with more or less intermediate (but nonetheless homogenous) methylation status in one of the groups. This can be seen in Supplementary Figures S4–S6, which show plots of the individual β-values of the top 10 significant CpGs in the two groups. The phenomenon was also statistically confirmed by comparing the mean difference (delta) of M-values for the top 100 CpGs from all contrasts by ANOVA and pairwise t-tests (adjusted *p*-value < 0.001 for all comparisons; Figure 4).

The comparison between MMC1 and NMC resulted in 597 significant DMRs, with a medium size of 1350 bp (99–10,750 bp). The comparison between MMC2 and NMC resulted in much fewer (63) DMRS, with a median size of 1307 bp (145–3026 bp). An intermediate number of 128 DMRs, with a median size of 1255 bp (199–7186 bp), was called when comparing MMC1 vs. MMC2. The top ten DMRs from each comparison were evaluated with the UCSC genome browser [15] to identify overlapping gene transcription start sites or regulatory DNA elements such as gene promoters or enhancers. The top DMRs of MMC1 compared with the other two groups were quite similar and mostly encompassed regulatory elements such as miRNA clusters (e.g., on chromosome 14), gene promoters, or the *PRAME* gene on chromosome 22 (Table 2).

## 4. Discussion

Our study investigated the genetic and epigenetic landscape of CM and MN. During cohort selection, we specifically aimed to include as many CM cases with a TWT genotype as possible to compare their genetic and epigenetic landscape to the more common MAPK-mutated cases. As a result, the cohort does not reflect the typical distribution of CM subtypes and locations but is enriched for acrally localized tumors, particularly ALM. The main limitation of the cohort is the relatively small sample size, with 19 CM and 11 MN cases. Although a larger number of samples (56 per entity) were initially collected, many had to be excluded due to failing the strict quality and biological requirements for DNA samples used for methylome analysis.

The majority of excluded samples had insufficient DNA yield or showed severe degradation. This is largely due to the retrospective nature of the study, as many of the archived FFPE tissue blocks were up to or over a decade old. Supplementary Figure S1 clearly illustrates that the excluded samples were significantly older than the samples included in the final cohort. The bias toward larger, more advanced tumors also stems from this issue, as these lesions usually provide higher DNA yields. Since fresh tissue generally allows for better DNA quality, future studies should prioritize a multicenter approach focusing on recently diagnosed patients rather than relying on retrospective recruitment limited to only a few local centers.

Another major reason for sample exclusion was the general requirement for high tumor cell content for methylome analysis [35]. To retain as many samples as possible without compromising data integrity, we combined a bioinformatic estimation of tumor purity with an assessment of whether samples clustered distinctly or not. Despite this effort, our approach using the described methods was not able to fully resolve the inherent bias towards samples with high tumor cell contents. CM subtypes with a less dense growth pattern remained underrepresented in the final cohort. Future advances in technology may help address this issue. Methods such as long-read sequencing [36] and artificial intelligence-driven models (e.g., similar to the concept described by Yasumizu et al., 2024 [37]) could enable the analysis of mixed tissues and samples with low tumor cell content in the future, allowing for a more comprehensive view of CM heterogeneity.

The genetic findings in CM—both at molecular and cytogenomic levels—match the described patterns in the literature. The distribution of driver mutations in our study is admittedly slightly skewed compared to the frequencies reported in the literature [2,38]. However, this can be accounted for by the selection bias during the generation of the cohort and the subsequent overrepresentation of cases with a TWT status. CM with one of the three MAPK-activating driver mutations presented with a high mutational burden combined with a heavily distorted genome structure with several or many extensive CNVs. TWT-CMs, on the other hand, had lower mutational burden and a CNV profile shifting towards more focal abnormalities. *TERT* promoter mutations are common alterations in CM, found in approximately 70% of cases [29,39]. In our study, *TERT* promoter mutations were highly common in MAPK-altered CM but much rarer in TWT cases. This is in accordance with previous reports, which found that *TERT* promoter mutations are rare events in acral CMs [40,41,42], which represent the majority of TWT cases in our study. The differences between the two groups may be even more pronounced if one re-classifies these cases into an MAPK-altered group with (rare) alterations in other MAPK-associated genes such as *MAP2K1* mutations [43], *KIT* alterations [44], *BRAF* gene fusions [45], or other. However, as only a part of those alterations can be reliably detected with the utilized methods, we decided to use the traditional definition established by The Cancer Genome Atlas in 2015 [16]. MN showed—as expected—only a few mutations in addition to the recognized driver mutations (mostly *BRAF* p.V600) and lacked both CNVs and *TERT* promoter mutations.

Dimensional reduction of DMA resulted in a clear separation of CM and MN cases into two methylation clusters. Within the CM cohort, no differences between CM with MAPK-affecting driver mutations and cases with TWT status were found. This is similar to the findings of Jurmeister et al., 2021 [46], who did not detect major differences in the methylome structure when comparing mucosal melanoma (with a high frequency of cases without MAPK driver mutation) and CM. The exception was one single sample, which localized separately from all other CM specimens. This sample additionally showed a unique genetic pattern, with a low mutational burden and only a few but focal CNV abnormalities. Inclusion of an external dataset published by Pradhan et al., 2019 [4] confirmed the existence of a second methylation cluster including this sample. In the external cohort, a higher number of samples belonged to the MMC2 cluster compared to our internal cohort. The majority of them were classified as ALM by the authors. However, no further information about the single sample was provided; therefore, we were not able to assess whether there is a distinct histological or molecular marker that connects these cases.

An in-depth analysis of the three identified methylation clusters revealed extensive differences between the three groups. The top DMRs of MMC1, in comparison to the other two groups, were quite similar and mostly encompassed regulatory elements such as miRNA clusters and gene promoters. When compared to NMC, one of the top hypomethylated DMRs overlapped with the *PRAME* gene. As the full name “Preferentially Expressed in Melanoma” indicates, this tumor marker is expressed in CM but only rarely in MN [47].This is in accordance with the distinct hypomethylation of the gene region detected in our study. Markers in other regions, such as the *CCDC140*/*PAX3* region on chromosome 2, are in accordance with a previously published study by Conway et al., 2022 [11]. Both comparisons involving MMC2 identified a high proportion of CpGs with intermediate methylation levels.

Despite the relatively small cohort due to the high sample dropout rate, our study demonstrates that DMA combined with dimensional reduction methods such as UMAP can robustly distinguish CM from MN. However, all investigated lesions can be classified as benign or malignant using standard histology. The more intriguing question is how borderline lesions, such as dysplastic, Spitz, or Reed nevi, behave in this approach and whether their DNA methylation patterns contain information about malignant potential. Furthermore, our study identified a possible new molecular CM subtype with a distinct DNA methylation profile. Since most of these samples originated from an external cohort without additional metadata, the commonality underlying this subtype remains unclear. Larger studies focusing on uncommon melanocytic lesions are needed to address this question.

In the future, DNA methylome analysis may be a promising tool for enhancing diagnostic and prognostic accuracy in the clinical workup of melanocytic lesions. While the results of this study should be interpreted with caution, they provide a solid basis for further large, prospective, and independent studies to validate the clinical utility of the potential epigenetic biomarkers identified here.

## Figures and Tables

**Table 1:** General overview of the clinical characteristics of the samples included.

Variable	*n* (%) or Mean ± SD
**Gender**	
Cutaneous melanoma	
Female	10 (53%)
Male	9 (47%)
Melanocytic nevi	
Female	5 (45%)
Male	6 (55%)
Skin	
Female	6 (55%)
Male	5 (45%)
**Age at diagnosis (years, mean ± SD)**	
Cutaneous melanoma	59.8 ± 23.8 (31–96)
Melanocytic nevi	34.1 ± 11.9 (16–52)
**Altitude of residence (cutaneous melanoma)**	
<1000 m	16 (84%)
>1000 m	3 (16%)
**Localization**	
Cutaneous melanoma	
Head or neck	3 (15%)
Trunk	4 (21%)
Extremities	6 (32%)
Acral	6 (32%)
Melanocytic nevi	
Head or neck	3 (27%)
Trunk	6 (55%)
Extremities	2 (18%)
**Histological subtype**	
Cutaneous melanoma	
Desmoplastic melanoma	1 (5.25%)
Acral lentiginous melanoma	4 (21%)
Lentigo maligna melanoma	1 (5.25%)
Nodular melanoma	8 (42%)
Spindle cell melanoma	2 (11%)
Superficial spreading melanoma	3 (16%)
Melanocytic nevi	
Compound nevus	7 (64%)
Dermal nevus	4 (36%)
**Tumor stage (cutaneous melanoma)**	
IIA	1 (5.25%)
IIB	3 (16%)
IIC	4 (21%)
III	1 (5.25%)
IIIA	1 (5.25%)
IIIB	1 (5.25%)
IIIC	5 (26%)
IV	3 (16%)

**Table 2:** Differentially methylated clusters. The top 10 hits for each comparison (MMC1 vs. NMC, MMC2 vs. NMC, and MMC1 vs. MMC2) are shown. The regions are ordered according to their significance (see Supplementary Data for more details). The last column shows the location of overlapping gene transcription start sites and GeneHancer (double elite) promoter or enhancer elements. Methylation status is depicted as + for hypermethylated and – for hypomethylated.

Location	Range	Length	Meth. Status	DNA Elements of Interest
MMC1 vs. NMC				
chr2	223,163,573–223,172,329	8757	+	*CCDC140*, *PAX3*
chr14	10,1505,130–101,515,879	10,750	−	microRNA cluster
chr3	147,122,664–147,131,860	9197	+	*ZIC4*, *ZIC1*, GH03J147407
chr14	101,487,756–101,493,252	5497	−	microRNA cluster
chr2	200,328,645–200,336,146	7502	+	*ATB2*, *SAT2B*, GH02J199454
chr6	29,520,527–29,521,803	1277	+	*OR2I1P*, GH06J029552
chr22	22,898,356–22,902,665	4310	−	*PRAME*
chr3	157,812,018–157,817,678	5661	+	SHOX2, GH03J158097
chr6	31,650,735–31,651,676	942	+	GH06J031682
chr14	60,972,853–60,978,852	6000	+	*SIX6*
MMC2 vs. NMC				
chr3	46,446,998–46,449,636	2639	−	*CCR5AS*, *CCRL2*, GH03J046404
chr1	160,680,856–160,682,655	1800	−	*CD48*, GH01J160703
chr1	233,248,709–233,249,314	606	−	-
chr13	102,568,345–102,570,482	2138	−	*FGF14*
MMC2 vs. NMC (*continued*)				
chr2	176,963,315–176,965,729	2415	+	*HOXD12*
chr1	203,320,190–203,321,087	898	−	GH01J203319
chr14	61,108,227–61,110,649	2423	+	*SIX1*, GH14J060640
chr18	53,068,921–53,070,851	1931	−	*TCF4*, GH18J055398
chr11	2,846,681–2,848,492	1812	−	GH11J002824
chr1	234,907,722–234,908,514	793	−	GH01J234766
MMC1 vs. MMC2				
chr14	101,487,756–101,493,252	5497	−	microRNA cluster
chr2	166,649,910–166,651,571	1662	+	*GALTN3*, GH02J165791
chr14	101,518,766–101,522,431	3666	−	microRNA cluster
chr7	157,527,573–157,534,758	7186	−	-
chr1	203,320,190–203,321,854	1665	+	*FMOD*, GH01J203349
chr10	106,027,915–106,029,358	1444	+	*MIR4428*, *STO2*, *GSTO2*, GH10J104267
chr12	120,241,287–120,242,513	1227	+	GH12J119803
chr2	54,784,402–54,786,148	1747	+	*SPTBN1*, GH02J05455
chr1	234,667,087–234,668,366	1280	+	*LINC01354*, GH01J234527
chr1	91,300,215–91,302,117	1903	+	LINC02609

## Data Availability

The microarray datasets generated during the current study are available in the ArrayExpress repository, E-MTAB-14045. All other relevant datasets can be found in the Supplementary Data File.

## Supplementary Materials

The following supporting information can be downloaded at: https://www.mdpi.com/article/10.3390/diagnostics15050531/s1, Figure S1: Comparison of FFPE block age of included and excluded samples. (a) Box-and-Whiskers plot depicting the distribution of block age (in years) of excluded/not included (blue) and included (green) BN samples. (b) Simultaneous plot depicting the distribution of block age (in years) of excluded (violet) and included (mustard) CM specimens. For both comparisons, the block age of excluded samples is significantly higher as determined by the Wilcoxon test (*p*-value: <0.001 for BN and *p*-value: 0.005 for CM); Figure S2: Biological parameters of excluded and included samples. (a–c) Box-and-Whiskers plot showing tumor size, Breslow’s depth, and cell vitality of excluded (violet) and included (mustard) CM samples. (d) Bar plot of ulceration status of excluded (violet) and included (mustard) CM specimens. (e) Distribution of excluded/not included (violet) and included (mustard) CM samples according to their tumor stage depicted as bar plots; Figure S3: Methylation clusters determined by UMAP reduction analysis of the internal cohort. Two-dimensional UMAP reduction plot of all samples. Each dot represents the overall methylome structure of one sample. UMAP reduction was performed on the EpiDip server together with approximately 25,000 other cancer and normal samples. The coordinates of the specimens in this study were subsequently extracted. The individual methylation clusters are called melanoma methylation cluster 1 (MMC1; green), melanoma methylation cluster 2 (MMC2; magenta), nevus methylation cluster (NMC; orange), and skin methylation cluster (SMC; blue). Figures S4–S6: Visualization of the top 20 differentially methylated CpGs in pairwise cluster comparisons. The individual dots in each plot represent the β-value (0 = unmethylated; 1 = methylated) of each sample in the respective groups; Table S1: Overview of the covered regions of the used custom sequencing panel; Supplementary Data.
