# Supplementary material for: DNA Methylation Array Analysis Identifies Biological Subgroups of Cutaneous Melanoma and Reveals Extensive Differences with Benign Melanocytic Nevi
Source: Diagnostics (Basel). 2025 Feb 21;15(5):531. doi: 10.3390/diagnostics15050531 (PMC11899029; doi:10.3390/diagnostics15050531)
Supplement: Supplementary file 1 [file diagnostics-15-00531-s001.zip › Supplementary Figure Legends.docx]

**Supplementary Figure S1: Comparison of FFPE block age for included and excluded samples. a** Box-and-Whiskers plot depicting the distribution of block age in years of excluded/not included (blue) and included (green) BN samples. **b** Simultaneous plot depicting the distribution of block age in years for excluded (violet) and included (mustard) CM specimen. For both comparisons, the block age of excluded samples is significantly higher as determined by Wilcoxon test (p-value: < 0.001 for BN and p-value: 0.005 for CM).

**Supplementary Figure S2: Biological Parameters of excluded and included samples. a-c** Box-and-Whiskers plot showing tumor size, Breslow’s depth and cell vitality for excluded (violet) and included (mustard) CM samples. **d** Bar plot of ulceration status of excluded (violet) and included (mustard) CM specimen. **e** Distribution of excluded/not included (violet) and included (mustard) CM samples according to their tumor stage depicted as bar plot.

**Supplementary Figure S3: Methylation clusters determined by UMAP reduction analysis for internal cohort.** Two-dimensional UMAP reduction plot of all samples from the internal cohort. Each dot represents the overall methylome structure of one sample. UMAP reduction was performed on the EpiDip server together with approximately 25 000 other cancer and normal samples. The coordinates of the specimen of this study were subsequently extracted. The individual methylation clusters are called melanoma methylation cluster 1 (MMC1; green), melanoma methylation cluster 2 (MMC2; magenta), nevus methylation cluster (NMC; orange) and skin methylation cluster (SMC; blue).

**Supplementary Figure S4: Visualization of the top 20 differentially methylated CpGs in MMC1 compared to NMC.** The individual dots in each plot represents the β-value (0 = unmethylated; 1 = methylated) of each sample from the respective groups.

**Supplementary Figure S5: Visualization of the top 20 differentially methylated CpGs in MMC2 compared to NMC.** The individual dots in each plot represents the β-value (0 = unmethylated; 1 = methylated) of each sample from the respective groups.

**Supplementary Figure S6: Visualization of the top 20 differentially methylated CpGs in MMC2 compared to MMC1.** The individual dots in each plot represents the β-value (0 = unmethylated; 1 = methylated) of each sample from the respective groups.
