# Supplementary material for: DNA Methylation Array Analysis Identifies Biological Subgroups of Cutaneous Melanoma and Reveals Extensive Differences with Benign Melanocytic Nevi
Source: Diagnostics (Basel). 2025 Feb 21;15(5):531. doi: 10.3390/diagnostics15050531 (PMC11899029; doi:10.3390/diagnostics15050531)
Supplement: Supplementary file 1 [file diagnostics-15-00531-s001.zip › Table S1.docx]

**Supplementary Table S1:** **Overview of the covered regions of the used custom sequencing panel.** The common gene names, chromosomal localization, corresponding RefSeq transcripts (hg19) and the target regions are listed. Genes especially relevant for CM are marked in bold.

| **Number** | **Gene Name** | **Localization** | **RefSeq Transcript** | **Covered Regions** |
| --- | --- | --- | --- | --- |
| 1 | *ABL1* | 9q34.1 | NM_005157.5 | e4 - e9 |
| 2 | *AKT1* | 14q32.33 | NM_005163 | e3 |
| 3 | *ALK* | 2p23.2-p23.1 |  | full |
| 4 | *APC* | 5q22.2 |  | full |
| 5 | *ARID1A* | 1p36.11 |  | full |
| **6** | ***ARID2*** | **12q12** |  | **full** |
| 7 | *ASXL1* | 20q11.1 | NM_015338.5 | e13 |
| 8 | *ATM* | 11q22.3 |  | full |
| 9 | *ATR* | 3q23 |  | full |
| 10 | *ATRX* | Xq21.1 |  | full |
| 11 | *B2M* | 15q21.1 |  | full |
| **12** | ***BAP1*** | **3p21.1** |  | **full** |
| 13 | *BARD1* | 2q35 |  | full |
| 14 | *BCL2* | 18q21.33 | NM_000633 | full |
| 15 | *BCOR* | Xp11.14 |  | full |
| 16 | *BCORL1* | Xq26.1 |  | full |
| 17 | *BIRC3* | 11q22.1 |  | full |
| **18** | ***BRAF*** | **7q34** | **NM_004333.5** | **from e8** |
| 19 | *BRCA1* | 17q21.31 |  | full |
| 20 | *BRCA2* | 13q13.1 |  | full |
| 21 | *BRIP1* | 17q23.2 |  | full |
| 22 | *BTK* | Xq22.1 | NM_000061.2 | e11, e15, e16 |
| 23 | *CALR* | 19p13.13 | NM_004343.3 | e9 |
| 24 | *CARD11* | 7p22.2 |  | full |
| 25 | *CBL* | 11q23.3 | NM_005188.3 | e8 - e9 |
| **26** | ***CCND1*** | **11q13.3** |  | **full, 5'-UTR** |
| 27 | *CCND2* | 12p13.32 |  | full |
| 28 | *CCND3* | 6p21.1 |  | full |
| 29 | *CD79B* | 17q23.3 |  | full |
| 30 | *CDH1* | 16q22.1 |  | full |
| 31 | *CDK12* | 17q12 |  | full |
| 32 | *CDK4* | 12q14.1 |  | full |
| **33** | ***CDKN2A*** | **9p21.3** |  | **full** |
| 34 | *CEBPA* | 19q13.1 |  | full |
| 35 | *CHEK2* | 22q12.1 |  | full |
| 36 | *CREBBP* | 16p13.3 |  | full |
| 37 | *CSF3R* | 1p34.3 | NM_156039.3 | e14 - e17 |
| 38 | *CTNNB1* | 3p22.1 |  | full |
| 39 | *CUX1* | 7q22.1 |  | full |
| 40 | *CXCR4* | 2q22.1 |  | full |
| **41** | ***DDX3X*** | **Xp11.4** |  | **full** |
| 42 | *DIS3* | 13q21.3 |  | full |
| 43 | *DNMT3A* | 2p23.3 |  | full |
| 44 | *EGFR* | 7p11.2 | NM_00528 | full |
| 45 | *EGR1* | 5q31.2 |  | full |
| 46 | *EP300* | 22q13.2 |  | full |
| 47 | *EPCAM* | 2p21 |  | full |
| 48 | *ERBB2* | 17q12 |  | full |
| 49 | *ETNK1* | 12p12.1 | NM_018638.4 | e3 |
| 50 | *ETV6* | 12p13.2 |  | full |
| 51 | *EZH2* | 7q35-36 |  | full |
| 52 | *FAM46C* | 1p12 |  | full |
| 53 | *FBXW7* | 4q31.3 | NM_033632.3 | e8 - e12 |
| 54 | *FGFR2* | 10q26.13 |  | full |
| 55 | *FLT3* | 13q12 | NM_004119.2 | e14, e15, e20 |
| 56 | *FOXL2* | 3q22.3 |  | full |
| 57 | *FOXO1* | 13q14.11 |  | full |
| 58 | *GATA2* | 3q21.3 |  | full |
| **59** | ***GNA11*** | **19p13.3** | **NM_002067.5** | **e5** |
| 60 | *GNA13* | 17q24.1 |  | full |
| **61** | ***GNAQ*** | **9q21.2** | **NM_002072.5** | **e1 - e5** |
| 62 | *GNAS* | 20q13.3 | NM_000516.4 | e8, e9 |
| 63 | *H3F3A* | 1q42.12 |  | full |
| 64 | *HIST1H1B* | 6p22.1 |  | full |
| 65 | *HOXD8* | 2q31.1 |  | full |
| **66** | ***HRAS*** | **11p15.5** | **NM_005343.4** | **e2 - e4** |
| 67 | *ID3* | 1p36.12 |  | full |
| **68** | ***IDH1*** | **2q34** | **NM_005896.3** | **e4** |
| 69 | *IDH2* | 15q26.1 | NM_002168.3 | e4 |
| 70 | *IKZF1* | 7p12.2 |  | full |
| 71 | *JAK1* | 1p31.3 | NM_002227.4 | e16, e20 - e24 |
| 72 | *JAK2* | 9p24 |  | full |
| 73 | *JAK3* | 19p13.11 |  | full |
| 74 | *KDM6A* | Xp11.3 |  | full |
| **75** | ***KIT*** | **4q12** |  | **full** |
| 76 | *KLF2* | 19p13.11 |  | full |
| 77 | *KLHL6* | 3q27.1 |  | full |
| 78 | *KMT2C* | 7q36.1 |  | full |
| 79 | *KMT2D* | 12q13.12 |  | full |
| **80** | ***KRAS*** | **12p12.1** | **NM_033360.3** | **e2 - e4** |
| 81 | *LZTR1* | 22q11.21 |  | full |
| 82 | *MAD2L1* | 4q27 |  | full |
| **83** | ***MAP2K1*** | **15q22.31** |  | **full** |
| 84 | *MAP2K2* | 19p13.3 |  | full |
| 85 | *MAP2K4* | 17p12 |  | full |
| 86 | *MED12* | Xq13.1 | NM_005120.2 | e2 |
| 87 | *MEF2B* | 19p13.11 |  | full |
| **88** | ***MET*** | **7q31** |  | **full** |
| 89 | *MITF* | 3p13 |  | full |
| 90 | *MLH1* | 3p22.2 |  | full |
| 91 | *MPL* | 1p34 |  | full |
| 92 | *MSH2* | 2p21-p16.3 |  | full |
| 93 | *MSH6* | 2p16.3 |  | full |
| 94 | *MYC* | 8q24.2 |  | full |
| 95 | *MYD88* | 3p22.2 |  | full |
| **96** | ***NF1*** | **17q11.2** |  | **full** |
| 97 | *NF2* | 22q12.2 |  | full |
| 98 | *NFE2* | 12q13.13 |  | full |
| 99 | *NFKBIE* | 6p21.1 |  | full |
| **100** | ***NOTCH1*** | **9q34.3** | **NM_017617.5** | **e26 - e28, e34, 3'-UTR** |
| 101 | *NOTCH2* | 1p12 | NM_024408.4 | e26 - e28, e34 |
| 102 | *NOTCH3* | 19p13.12 |  | full |
| 103 | *NPM1* | 5q35 | NM_002520.6 | e11 |
| **104** | ***NRAS*** | **1p13.2** | **NM_002524.4** | **e2 - e4** |
| 105 | *PALB2* | 16p12.2 |  | full |
| 106 | *PAX5* | 9p13.2 |  | full |
| 107 | *PDGFRA* | 4q12 |  | full |
| 108 | *PHF6* | Xq26.2 |  | full |
| 109 | *PIGA* | Xp22.2 |  | full |
| **110** | ***PIK3CA*** | **3q26.32** |  | **full** |
| 111 | *PLCG2* | 16q23.3 | NM_002661 | full |
| 112 | *PMS2* | 7p22.1 |  | full |
| 113 | *POLE* | 12q24.33 |  | full |
| 114 | *POT1* | 7q31.33 |  | full |
| 115 | *PPM1D* | 17q23.2 |  | full |
| **116** | ***PPP6C*** | **9q33.3** |  | **full** |
| 117 | *PRDM1* | 6q21 |  | full |
| **118** | ***PTEN*** | **10q23.31** |  | **full** |
| 119 | *PTPN11* | 12q24.1 |  | full |
| 120 | *PTPRD* | 9p24.1-p23 |  | full |
| **121** | ***RAC1*** | **7p22.1** | **NM_006908.5** | **e1 - e7** |
| 122 | *RAD21* | 8q24.11 |  | full |
| 123 | *RAD51C* | 17q22 |  | full |
| 124 | *RAD51D* | 17q12 |  | full |
| **125** | ***RASA2*** | **3q23** |  | **full** |
| **126** | ***RB1*** | **13q14.2** |  | **full** |
| 127 | *RET* | 10q11.21 | NM_020975 | e16 |
| 128 | *RHOA* | 3p21.31 | NM_001664.4 | e2 |
| 129 | *RUNX1* | 21q22.3 |  | full |
| 130 | *SETBP1* | 18q12.3 | NM_015559.2 | e4 |
| 131 | *SF3B1* | 2q33.1 | NM_012433.3 | e13 - 16 |
| 132 | *SGK1* | 6q23.2 |  | full |
| 133 | *SH2B3* | 12q24.12 |  | full |
| 134 | *SMC3* | 10q25.2 |  | full |
| 135 | *SOCS1* | 16p13.13 |  | full |
| 136 | *SPEN* | 1p36.2 |  | full |
| 137 | *SRSF2* | 17q25.1 | NM_003016.4 | e1 |
| 138 | *STAG2* | Xq25 |  | full |
| 139 | *STAT3* | 17q21.2 | NM_139276.2 | e20 - 21 |
| 140 | *STAT5B* | 17q21.2 | NM_012448.3 | e16 |
| 141 | *STK11* | 19p13.3 |  | full |
| 142 | *TCF3* | 19p13.3 | NM_001136139.4 | e18 |
| **143** | ***TERT*** | **5p15.33** |  | **full, gene promoter** |
| 144 | *TET2* | 4q24 |  | full |
| 145 | *TNFAIP3* | 6q23.3 |  | full |
| 146 | *TNFRSF14* | 1p36.32 |  | full |
| **147** | ***TP53*** | **17p13.1** |  | **full** |
| 148 | *TRAF3* | 14q32.3 |  | full |
| 149 | *U2AF1* | 21q22.3 | NM_006758.2 | e2, e6 |
| 150 | *UBR5* | 8q22.3 | NM_015902.5 | e58 |
| 151 | *WT1* | 1p13 | NM_024426.2 | e7- e9 |
| 152 | *XPO1* | 2p15 | NM_003400.3 | e15 - e16 |
| 153 | *ZRSR2* | Xp22.1 |  | full |
