# Supplementary figures and images for: DNA Methylation Array Analysis Identifies Biological Subgroups of Cutaneous Melanoma and Reveals Extensive Differences with Benign Melanocytic Nevi
Source: Diagnostics (Basel). 2025 Feb 21;15(5):531. doi: 10.3390/diagnostics15050531 (PMC11899029; doi:10.3390/diagnostics15050531)

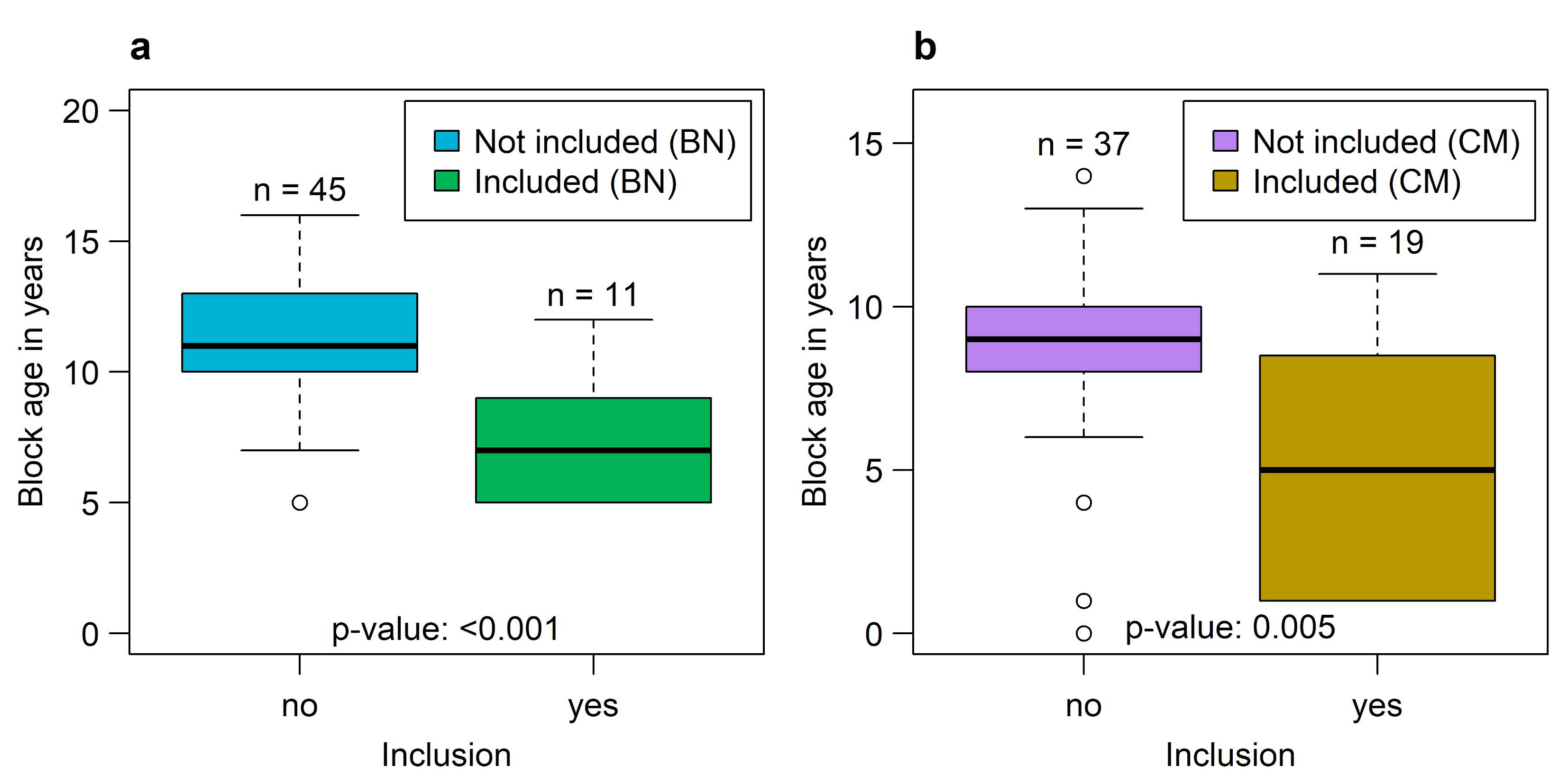

Supplement: Supplementary file 1 [file diagnostics-15-00531-s001.zip › Figure S1.png]

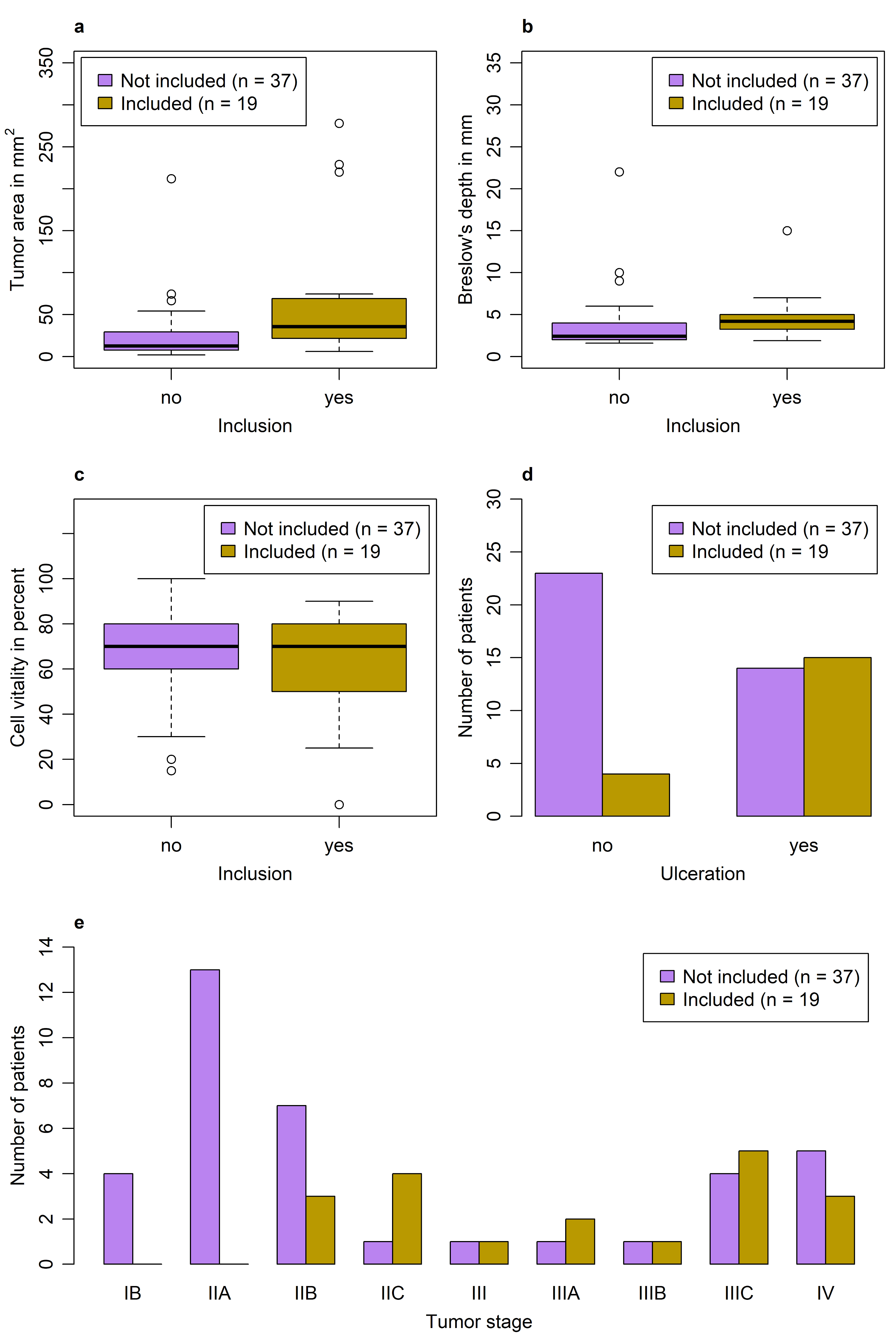

Supplement: Supplementary file 1 [file diagnostics-15-00531-s001.zip › Figure S2.png]

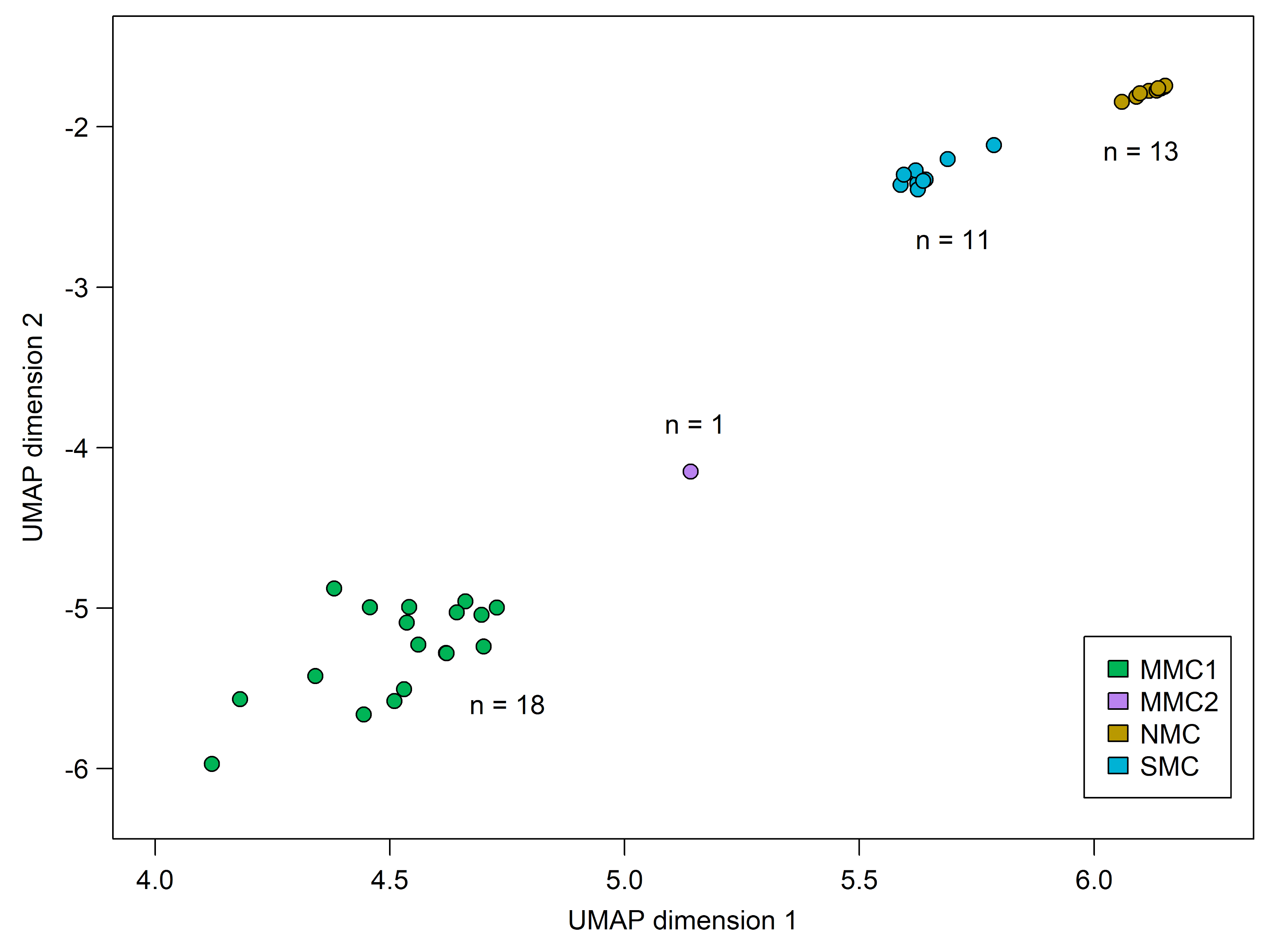

Supplement: Supplementary file 1 [file diagnostics-15-00531-s001.zip › Figure S3.png]

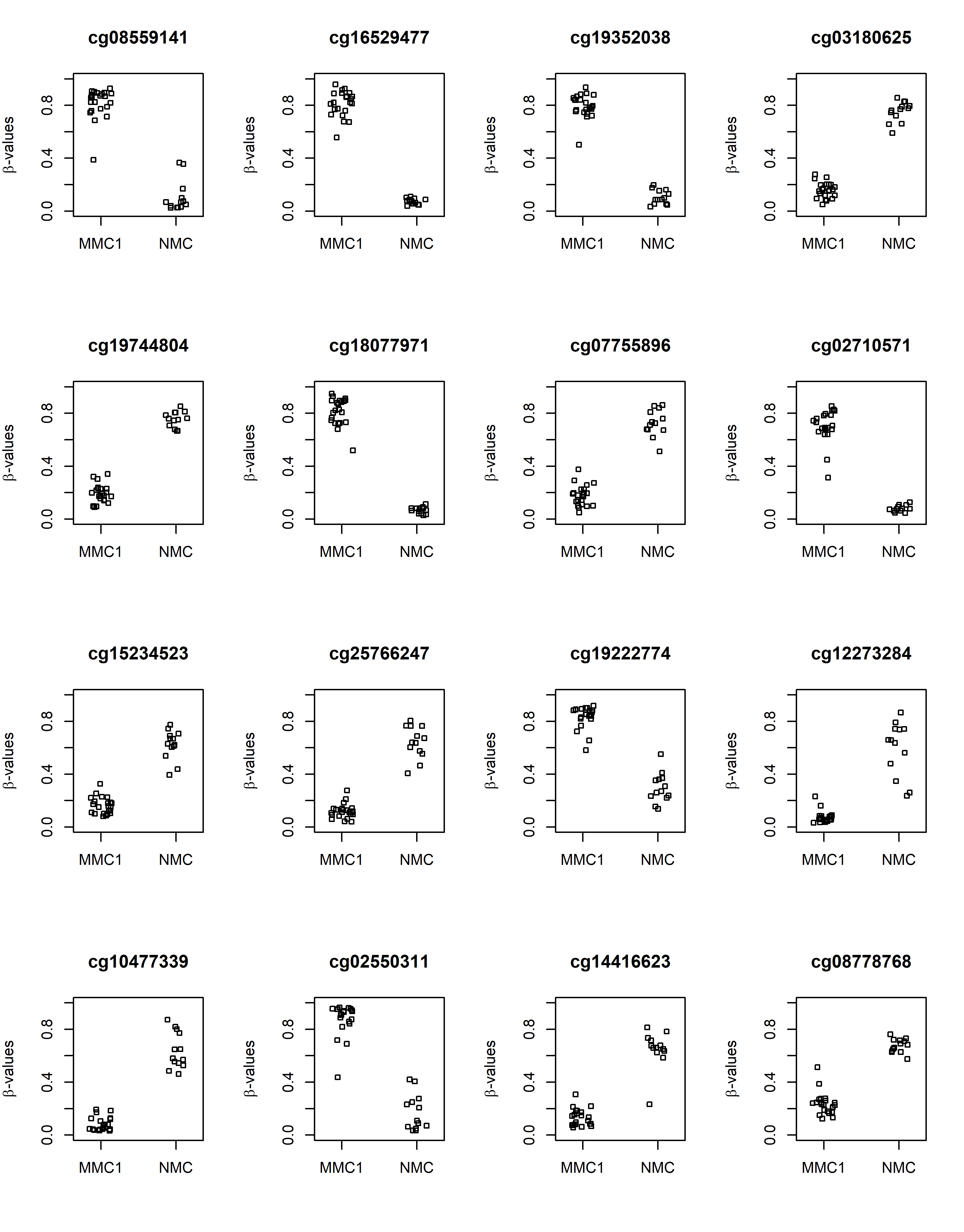

Supplement: Supplementary file 1 [file diagnostics-15-00531-s001.zip › Figure S4.png]

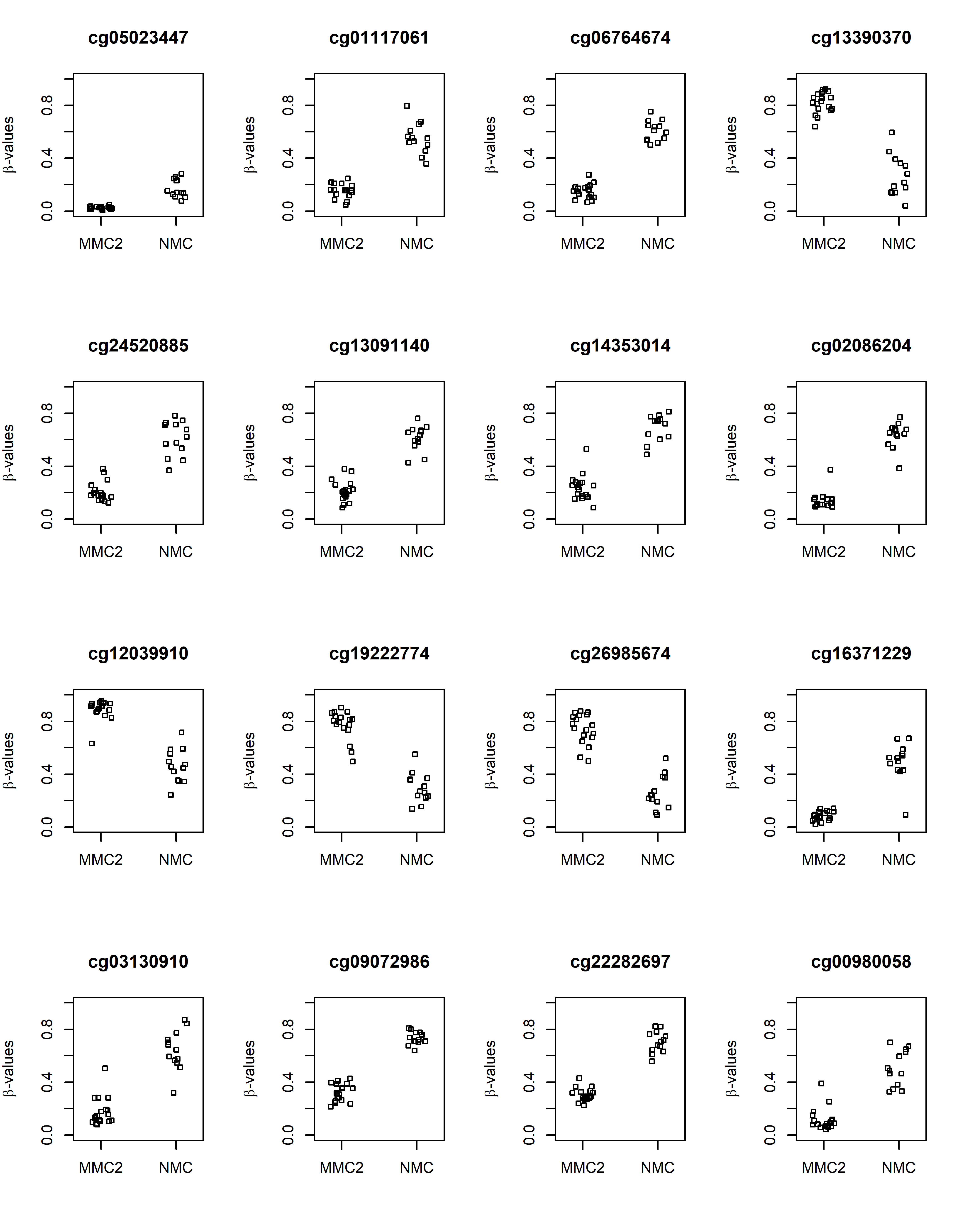

Supplement: Supplementary file 1 [file diagnostics-15-00531-s001.zip › Figure S5.png]

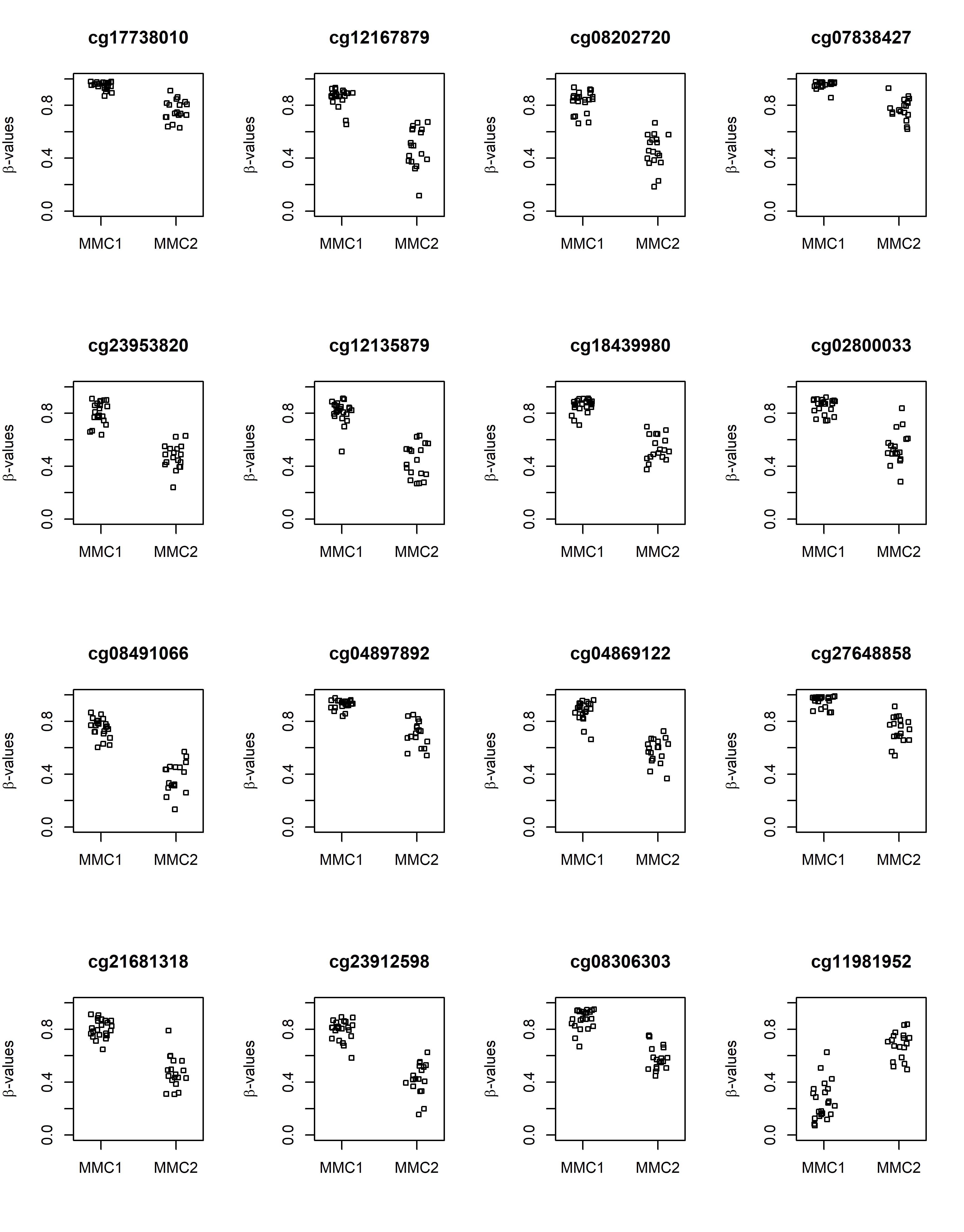

Supplement: Supplementary file 1 [file diagnostics-15-00531-s001.zip › Figure S6.png]
